# Growth Hormone Releasing Peptide-2 Attenuation of Protein Kinase C-Induced Inflammation in Human Ovarian Granulosa Cells

**DOI:** 10.3390/ijms17081359

**Published:** 2016-08-19

**Authors:** Yi-Ning Chao, David Sun, Yen-Chun Peng, Yuh-Lin Wu

**Affiliations:** 1 Department of Physiology, School of Medicine, National Yang-Ming University, Taipei 11221, Taiwan; stanley11306@hotmail.com; 2 Department of Obstetrics and Gynecology, Cheng Hsin General Hospital, Taipei 11221, Taiwan; shineway@hotmail.com; 3 Department of Internal Medicine, Taichung Veterans General Hospital, Taichung 40705, Taiwan; pychun@vghtc.gov.tw

**Keywords:** granulosa cell, inflammation, cyclooxygenase-2, interleukin-8, ghrelin, growth hormone releasing peptide-2

## Abstract

Cyclooxygenase-2 (COX-2) and interleukin-8 (IL-8) are two important inflammatory mediators in ovulation. Ghrelin may modulate inflammatory signaling via growth hormone secretagogue receptors. We investigated the role of ghrelin in KGN human ovarian granulosa cells using protein kinase C (PKC) activator phorbol 12, 13-didecanoate (PDD) and synthetic ghrelin analog growth hormone releasing peptide-2 (GHRP-2). GHRP-2 attenuated PDD-induced expression of protein and mRNA, the promoter activity of COX-2 and IL-8 genes, and the secretion of prostaglandin E2 (PGE_2_) and IL-8. GHRP-2 promoted the degradation of PDD-induced COX-2 and IL-8 proteins with the involvement of proteasomal and lysosomal pathways. PDD-mediated COX-2 production acts via the p38, c-Jun N-terminal kinase (JNK), extracellular signal-regulated kinase (ERK) and nuclear factor kappa-light-chain-enhancer of activated B cells (NF-κB) pathways; PDD-mediated IL-8 production acts via the p38, JNK and ERK pathways. GHRP-2 reduced the PDD-induced phosphorylation of p38 and JNK and activator protein 1 (AP-1) reporter activation and PDD-induced NF-κB nuclear translocation and reporter activation. The inhibitors of mitogen-activated protein kinase phosphatase-1 (MKP-1) and protein phosphatase 2 (PP2A) reduced the inhibitory effect of GHRP-2 on PDD-induced COX-2 and IL-8 expression. Our findings demonstrate an anti-inflammatory role for ghrelin (GHRP-2) in PKC-mediated inflammation of granulosa cells, at least in part, due to its inhibitory effect on PKC-induced activation of p38, JNK and NF-κB, possibly by targeting to MKP-1 and PP2A.

## 1. Introduction

Inflammation plays an important role in the host defense system that occurs in response to internal or external stimuli; it functions to counteract the insults exerted by these stimuli [1]. When acting as a finely tuned acute inflammation consequence that lasts for a short period of time, it is responsible for innate immunity and/or humoral immunity, and thus has therapeutic significance to the host. However, when inflammation becomes chronic and lasts for a long period of time, the outcome may have pathogenic consequences to the body [2].

Ovulation is a key event in the ovarian cycle that involves a number of regulatory networks. It is well-recognized that several aspects of ovulation resemble an inflammation-like reaction. Accumulated studies have demonstrated that various inflammatory factors are involved in ovulation. For example, cyclooxygenase-2 (COX-2) and interleukin-8 (IL-8) have been proposed to be of particular significance during ovulation [3,4]. The critical role of COX-2 in ovulation is well-established and this protein has been shown to regulate the production of prostaglandins (PGs), which are central to the ovulatory process [3]. Interleukin-8 is a chemotactic cytokine that acts to recruit and activate neutrophils and it is also regarded as an important player in ovulation [4,5].

The protein kinase C (PKC) family contains phospholipid-dependent serine/threonine kinases that are involved in a variety of physiological events. Activation of PKCs may lead to the activation of mitogen-activated protein kinases (MAPKs) or the targeting to the IκB kinase (IKK) complex, which results in nuclear factor kappa-light-chain-enhancer of activated B cells (NF-κB) activation, all of which are responsible for various inflammatory responses [6,7]. PKC signaling has been noted to regulate the expression of a number of inflammatory mediators, including COX-2 and IL-8 [8]. In fact, PKC signaling has been reported to participate in various ovarian activities, including follicular development, ovulation and luteolysis [9,10].

Ghrelin, a 28 amino acid peptide, was first identified in the stomach as an endogenous ligand for the growth hormone secretagogue receptor (GHSR) [11]. Growth hormone secretagogue is a small synthetic molecule that is able to stimulate the secretion of growth hormone from the pituitary [12]. Ghrelin receptor, GHSR, is a typical G protein-coupled receptor (GPCR) with seven transmembrane domains [13]. Two distinct ghrelin receptors can be expressed via splicing from a single GHSR gene, and the two proteins produced are GHSR type 1a (GHSR-1a) and GHSR-1b [14]. GHSR-1a is expressed in numerous different tissues and most prominently in several nuclei of the brain [15] and its expression has also been noted in ovary and testis [16,17]; GHSR-1b is mainly expressed in peripheral organs such as skin, liver, lung, spleen and kidney [18]. Recent studies have reported that not only both ghrelin and GHSR are expressed in the ovary [19], but also that GHSR-1a can be detected in both granulosa cells and theca cells during the human ovarian cycle [16]. In fact, blood ghrelin can be regularly detected in the women receiving in vitro fertilisation (IVF) procedure [20]; Ghrelin was previously shown to stimulate prolactin secretion in normal women [21]. The submaximal dose of ghrelin was not able to induce growth hormone secretion [22], but was able to suppress gonadotropin secretion in women [23]. Human ghrelin was also demonstrated to reduce the pituitary response to gonadotropin-releasing hormone (GnRH) in superovulated ewes [24]. Ghrelin was shown to improve the bovine blastocyst formation [25] and to enhance the maturation of bovine oocytes [26], but it was also reported to inhibit the GnRH-induced preovulatory gonadotropin surge in dairy heifers [27]. Accumulated findings from these studies indicate that ghrelin may play an important role in the ovarian system. In addition to growth hormone production and metabolism being regulated by ghrelin, previous studies have revealed that the plasma level of ghrelin is lower in patients suffering chronic inflammation [28]. This suggests that ghrelin may act as an anti-inflammatory factor. Ghrelin has a short half-life and therefore ghrelin analog growth hormone releasing peptide-2 (GHRP-2) has been extensively used in many studies. For example, administration of GHRP-2 has been shown to prevent the liver inflammatory response during endotoxemia [29]; GHRP-2 has also been demonstrated to attenuate endotoxin-induced IL-6 production and to decrease nitrite/nitrate release from peritoneal macrophages [30]. These findings suggest a novel anti-inflammatory function for ghrelin and GHRP-2 in physiology. Whether ghrelin is able to affect ovarian physiology by modulating the production of the various inflammatory players has not yet been extensively addressed. The aim of the current study was to characterize how the ghrelin analog GHRP-2 affects the various PKC-mediated inflammatory cascades in human granulosa cells by monitoring the COX-2/prostaglandin E2 (PGE_2_) and the IL-8 pathways, which are two critical components to be associated with inflammation and also known to be involved in ovarian physiology. This study also aimed to clarify the signaling mechanisms targeted by GHRP-2 that are involved in the PKC-regulated production of COX-2, PGE_2_ and IL-8.

## 2. Results

### 2.1. GHRP-2 Inhibition of the PKC-Induced Expression of COX-2 and IL-8 and Secretion of PGE_2_ and IL-8

**Figure 1:**
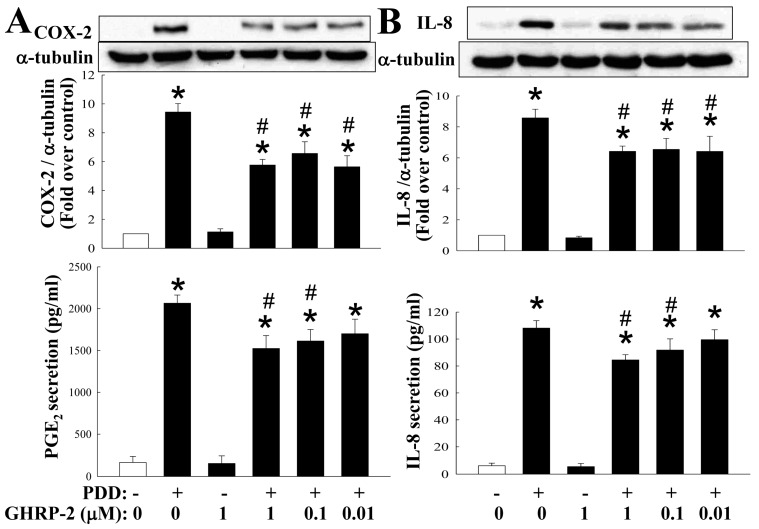
Growth hormone releasing peptide-2 (GHRP-2) inhibition of protein kinase C (PKC)-induced cyclooxygenase-2 (COX-2) and interleukin-8 (IL-8) expression and secretion of prostaglandin E2 (PGE_2_) and IL-8 in KGN cells. Overnight plated KGN cells were pretreated with GHRP-2 (0.01, 0.1, and 1 µM) for 2 h, and then phorbol 12,13-didecanoate (PDD) (100 nM) was included for an additional 12 h. The intracellular COX-2 (**A**) and IL-8 (**B**) protein expression levels and the resulting PGE_2_ (**A**) and IL-8 (**B**) protein concentrations in the cultured media were determined by Western blotting assay and ELISA, respectively. The results represent the means ± SEM (standard error of mean) (*n* = 4). * *p* < 0.05 compared with the control; ^#^ *p* < 0.05 compared with the PDD treatment.

To evaluate the role of ghrelin in ovarian inflammation, we used a PKC activator phorbol 12, 13-didecanoate (PDD) as a stimulus to treat KGN human ovarian granulosa cells in order to study whether the ghrelin analog GHRP-2 has an impact on the regulation of COX-2 and IL-8 expression. To ensure the specificity of the PKC-activation effect of PDD, a PKC inhibitor bisindolylmaleimide I (BIM I) was used. BIM I treatment at 1 and 5 µM significantly attenuated the induction of COX-2 and IL-8 expression by PDD (Figure S1). Before directly testing the role of GHRP-2 in PDD-induced inflammation, we first confirmed the presence of the cognate receptors for GHRP-2 in KGN cells by RT-PCR. It was found that GHSR-1a is constitutively expressed and its expression is not affected by either PDD or GHRP-2 (Figure S2); in contrast, the GHSR-1b was not detected. Having demonstrated specific PKC-mediated COX-2 and IL-8 expression using PDD and the presence of the GHRP-2 receptor GHSR-1a on the KGN cells, KGN cells were then pretreated with different doses of GHRP-2 (0.01, 0.1, and 1 µM) for 2 h before the inclusion of PDD (100 nM) for an additional 12 h of incubation. Expression of COX-2 and IL-8 protein, when induced by PDD, was attenuated by all doses of GHRP-2 (0.01, 0.1, and 1 µM) (Figure 1); GHRP-2 at 0.1 and 1 µM also was able to reduce the PDD-induced PGE_2_ and IL-8 secretion (Figure 1); GHRP-2 alone (1 µM) did not have any effect on the expression of COX-2 and IL-8 or the secretion of PGE_2_ and IL-8 (Figure 1).

**Figure 2:**
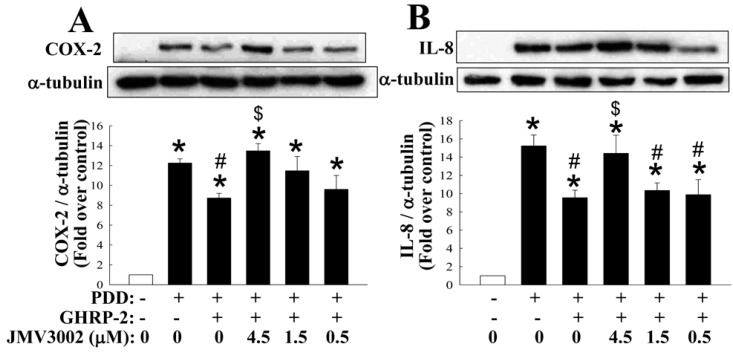
Specific effect of GHRP-2 on PKC-induced COX-2 and IL-8 protein expression. Plated KGN cells were pretreated with GHRP-2 (1 µM) in the absence or presence of the GHSR type 1a antagonist JMV3002 (0.5, 1.5, and 4.5 µM) for 2 h, and then PDD (100 nM) was included for an additional 12 h. The intracellular COX-2 (**A**) and IL-8 (**B**) protein expression levels were determined by Western blotting assay. The results represent the means ± SEM (*n* = 3). * *p* < 0.05 compared with the control; ^#^ *p* < 0.05 compared with the PDD treatment; ^$^ *p* < 0.05 compared with the combined GHRP-2 and PDD treatment.

To rule out the possibility that GHRP-2 has a cytotoxic effect on the KGN human ovarian granulosa cells used in this study, the viability indices of the KGN cells after the treatments outlined in Figure 1 were determined by alamarBlue and MTT assays. There was no apparent effect on the viability of the cells across all the treatments using either assay (Figure S3). To further confirm the specific effect of GHRP-2, KGN cells were pretreated with a GHSR-1a antagonist (JMV3002), and under this treatment the inhibitory effect of GHRP-2 on induction of COX-2 and IL-8 protein expression by PDD was reversed and the expression manners returned to levels that were comparable with the PDD alone treatment group (Figure 2), which suggests that GHRP-2 acts specifically via the GHSR-1a.

### 2.2. GHRP-2 Promotion of the Degradation of PKC-Induced COX-2 and IL-8 Proteins via Both Proteasomal and Lysosomal Pathways

**Figure 3:**
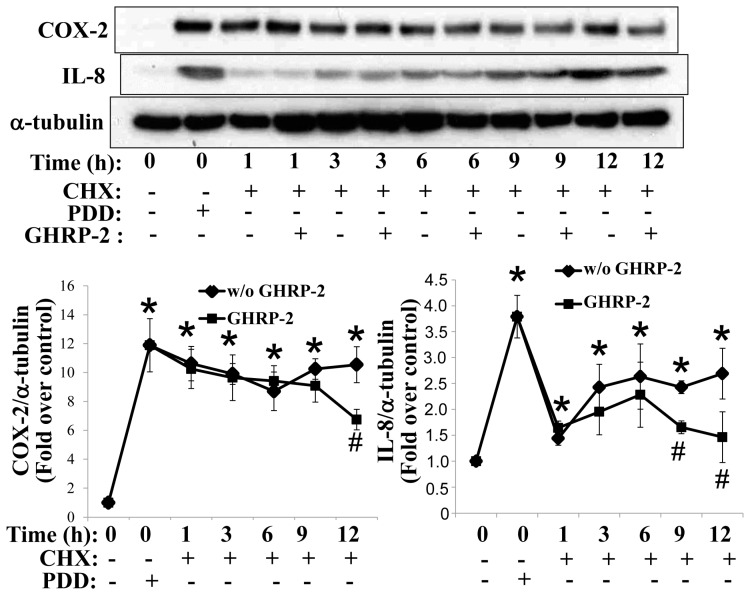
Promotion of the degradation of PKC-induced COX-2 and IL-8 proteins by GHRP-2. Plated KGN cells were either untreated or treated with PDD (100 nM) for 6 h to induce COX-2 and IL-8 protein expression (defined as 0 h), and then the cells were treated with cycloheximide (CHX, 5 µg/mL) or CHX in combination with GHRP-2 (1 µM) for 1, 3, 6, 9, and 12 h. The intracellular COX-2 and IL-8 protein expression levels were determined by Western blotting assay. The results represent the means ± SEM (*n* = 4) * *p* < 0.05 compared with the control; ^#^ *p* < 0.05 compared between with GHRP-2 and without GHRP-2 groups.

**Figure 4:**
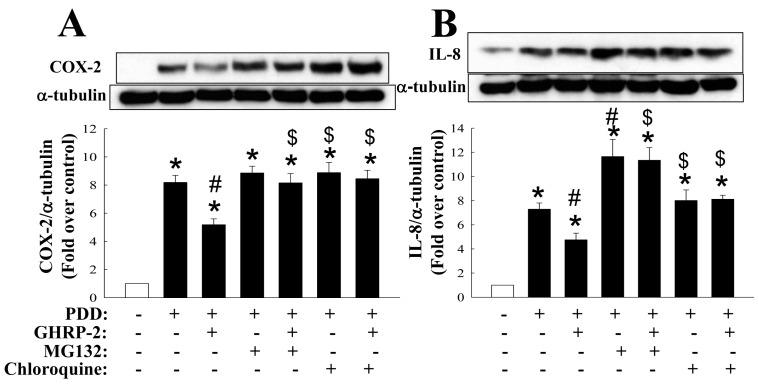
Involvement of the proteasome and lysosome pathways in the GHRP-2-enhanced degradation of COX-2 and IL-8 proteins. Plated KGN cells were pretreated with either the proteasome inhibitor MG132 (1 µM) or the lysosome inhibitor chloroquine (50 µM) in combination with GHRP-2 (1 µM) for 2 h, and then PDD (100 nM) was added for an additional 12 h. The intracellular COX-2 (**A**) and IL-8 (**B**) protein expression levels were determined by Western blotting assay. The results represent the means ± SEM (*n* = 4) * *p* < 0.05 compared with the control; ^#^ *p* < 0.05 compared with the PDD treatment; ^$^ *p* < 0.05 compared with the combined GHRP-2 and PDD treatment.

The GHRP-2 regulation of the PKC-mediated protein expression of COX-2 and IL-8 may occur at either the mRNA or the protein level. We first evaluated whether GHRP-2 was able to affect the stability of the PDD-induced COX-2 and IL-8 proteins. Cycloheximide (CHX, 5 µg/mL) was used to block de novo protein synthesis. It appeared that GHRP-2 was able to promote the degradation of PKC-induced COX-2 protein at 12 h and IL-8 protein at 9 h and 12 h (Figure 3). In this context, two protein degradation mechanisms, namely the proteasomal and the lysosomal proteolytic pathways, are well-recognized to regulate the turnover of a wide range of proteins [31]. Thus, KGN cells were pretreated with either a proteasome inhibitor MG132 (1 µM) or a lysosome inhibitor chloroquine (50 µM) in combination with GHRP-2 (1 µM), followed by PDD treatment (100 nM) for an additional 12 h. Both MG132 and chloroquine appeared to reverse the inhibitory effect of GHRP-2 on PDD-induced COX-2 and IL-8 protein expression (Figure 4). This supports the hypothesis that both the proteasomal pathway and the lysosomal pathway are involved in the promotion by GHRP-2 of the degradation of PDD-induced COX-2 and IL-8 proteins. Within the proteasomal degradation pathway there are a number of critical enzymes: ubiquitin-activating enzyme (E1), ubiquitin-conjugating enzyme (E2), and ubiquitin ligase (E3) [32]. In the ovary, a tumor suppressor gene BRCA1 has been shown to have ubiquitin E3 ligase activity and has been reported to be expressed in granulosa cells [33]. Within the lysosomal degradation pathway, an established lysosomal marker is cathepsin D, which has been detected in ovarian granulosa cells [34,35]. Based on the above findings, we next evaluated whether GHRP-2 is able to regulate BRCA1 and/or cathepsin D expression and thus mediate the degradation of the PDD-induced COX-2 and IL-8 proteins. It was not possible to detect BRCA1 in KGN cells; although cathepsin D was induced by PDD*;* nevertheless, GHRP-2 had no effect on cathepsin D expression (Figure S4).

### 2.3. GHRP-2 Attenuation of the Induction of COX-2 and IL-8 Transcription by PKC

**Figure 5:**
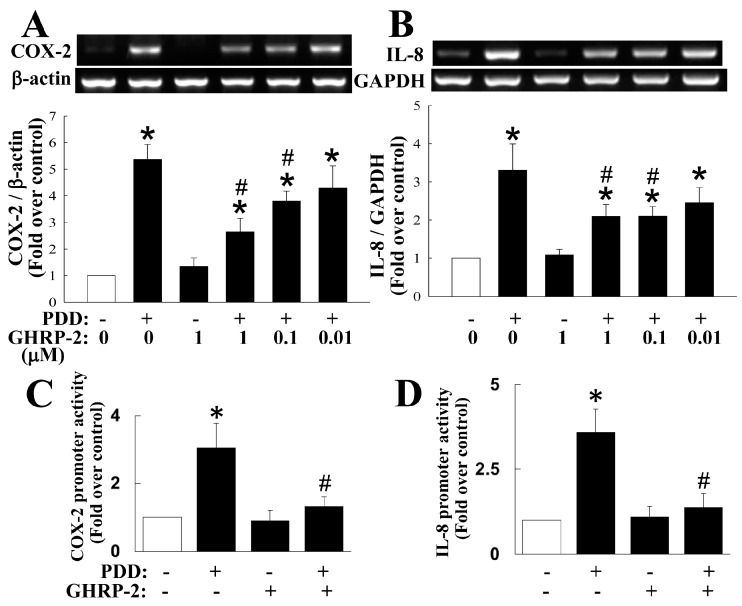
Attenuation of PKC-induced COX-2 and IL-8 transcription and mRNA expression by GHRP-2. Plated KGN cells were pretreated with GHRP-2 (0.01, 0.1, and 1 µM) for 2 h, and then PDD (100 nM) was included for an additional 12 h. The COX-2 (**A**) and IL-8 (**B**) mRNA expression levels were determined by RT-PCR. (**C**,**D**) To determine the promoter activity of COX-2 and IL-8 genes, plated KGN cells were transfected with a human COX-2 or a human IL-8 promoter construct, each with a luciferase reporter gene; these were co-transfected with pCMV-β-Gal as a control plasmid. The transfected cells were untreated, treated with either PDD (100 nM) or GHRP-2 (1 µM) alone or a combination of PDD and GHRP-2 for 12 h. Cells were then harvested with Glo lysis buffer and the COX-2 (**C**) or IL-8 (**D**) promoter activity was determined by luciferase assay; the luciferase activity was normalized against the β-galactosidase activity from pCMV-β-Gal within the same samples. The results represent the means ± SEM ((**A**,**B**): *n* = 4; (**C**,**D**): *n* = 3). * *p* < 0.05 compared with the control; ^#^ *p* < 0.05 compared with the PDD treatment.

Next we explored whether GHRP-2 is able to act at transcriptional level. Specifically, we examined the effect of GHRP-2 on induction of COX-2 and IL-8 mRNA expression by PDD using RT-PCR. The results showed that PDD is able to induce both COX-2 and IL-8 mRNA expression (Figure 5A,B). Furthermore, GHRP-2 at 0.1 and 1 µM apparently attenuated PDD-induced COX-2 and IL-8 mRNA expression (Figure 5A,B). GHRP-2 alone had no effect on the expression of either mRNA (Figure 5). To further clarify whether the regulation of COX-2 and IL-8 mRNA expression by PDD and GHRP-2 occurs at transcriptional level, we examined the transcription activity of the COX-2 and IL-8 promoter constructs in KGN cells. PDD alone appeared to induce both COX-2 and IL-8 promoter activation (Figure 5C,D), while GHRP-2 (1 µM) significantly inhibited PDD-mediated COX-2 and IL-8 promoter activation (Figure 5C,D); GHRP-2 alone did not affect either COX-2 or IL-8 promoter activity (Figure 5C,D).

### 2.4. Inhibition by GHRP-2 of PKC-Mediated Activation of Various MAPKs and NF-κB

**Figure 6:**
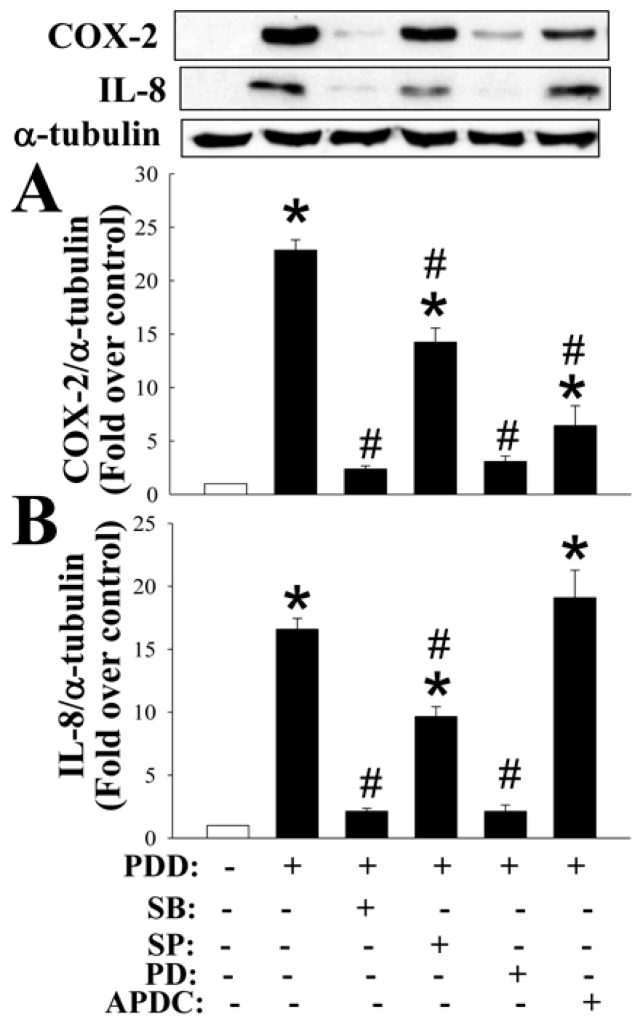
Identification of the signaling pathway(s) mediating PKC-induced COX-2 and IL-8 expression. Plated KGN cells were pretreated with mitogen-activated protein kinases (MAPKs) inhibitors individually (SB: p38 inhibitor, 20 µM; SP: c-Jun N-terminal kinase (JNK) inhibitor, 40 µM; PD: extracellular signal-regulated kinase (ERK) inhibitor, 20 µM), or nuclear factor kappa-light-chain-enhancer of activated B cells (NF-κB) inhibitor ammonium pyrrolidinedithiocarbamate (APDC, 50 µM) for 1 h and then PDD (100 nM) was included for an additional 12 h. The intracellular COX-2 (**A**) and IL-8 (**B**) protein expression levels were assessed by Western blotting assay. The results represent the means ± SEM (*n* = 4) * *p* < 0.05 compared with the control; ^#^ *p* < 0.05 compared with the PDD treatment.

**Figure 7:**
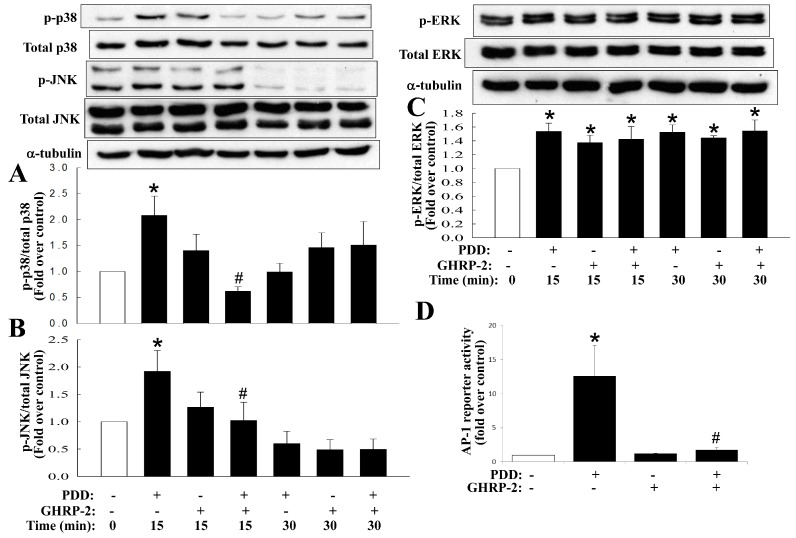
Attenuation of PKC-induced p38 and JNK phosphorylation and PKC-mediated activator protein 1 (AP-1) reporter activity by GHRP-2. Plated KGN cells were pretreated with GHRP-2 for 10 min and then the cells were exposed to PDD (100 nM) for 15 or 30 min. The effects of GHRP-2 on p38 (**A**); JNK (**B**); and ERK (**C**) phosphorylation were monitored by Western blotting assay; (**D**) To examine the impact of GHRP-2 on PKC-induced AP-1 reporter activation, plated KGN cells were transfected with an AP-1 reporter plasmid that controlled a luciferase reporter gene and co-transfected with pCMV-β-Gal as a control plasmid. Transfected cells were untreated, treated with either PDD (100 nM) or GHRP-2 (1 µM) alone, or treated with a combination of PDD and GHRP-2 for 12 h. The AP-1 reporter activity was determined by luciferase assay and normalized against the β-galactosidase activity from pCMV-β-Gal of the same samples. The results represent the means ± SEM ((**A**): *n* = 5; (**B**–**D**): *n* = 4) separate experiments. * *p* < 0.05 compared with the control; ^#^ *p* < 0.05 compared with the PDD treatment.

**Figure 8:**
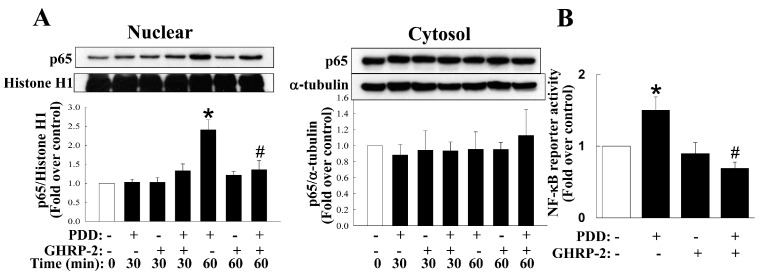
Inhibition of PKC-induced NF-κB nuclear translocation and activation by GHRP-2. (**A**) Plated KGN cells were pretreated with GHRP-2 for 10 min before the inclusion of PDD (100 nM) for additional 30 or 60 min. The amount of nuclear and cytosolic NF-κB p65 subunit present in these fractions was monitored by Western blotting assay. (**B**) To monitor NF-κB reporter activity, plated KGN cells were transfected with a plasmid bearing NF-κB responsive elements fused with a luciferase reporter gene and co-transfected with the pCMV-β-Gal as a control plasmid. Transfected cells were untreated, treated with either PDD (1 µM) or GHRP-2(1 µM) alone or treated with a combination of PDD and GHRP-2 for 12 h. The NF-κB reporter activity was determined by luciferase assay and normalized against the β-galactosidase activity from pCMV-β-Gal of the same samples. The results represent the means ± SEM ((**A**): *n* = 4; (**B**): *n* = 3) separate experiments. * *p* < 0.05 compared with the control; ^#^ *p* < 0.05 compared with the PDD treatment.

Previous studies have reported that the various MAPKs and NF-κB may mediate the expression of various inflammatory molecules [36]. Thus, inhibitors of various MAPKs (p38, JNK, and ERK) and NF-κB were used to clarify the signaling pathways post PKC activation. PDD-mediated COX-2 expression was found to be suppressed by inhibitors of p38 (SB2030580), JNK (SP600125), ERK (PD98059) and NF-κB (APDC) (Figure 6); PDD-mediated IL-8 expression was suppressed by inhibitors of p38, JNK and ERK (Figure 6). It has been noted in many cell types that PKC is able to activate the MAPKs as well as the NF-κB signaling pathways, namely the PKC-induced activation (phosphorylation) of MAPKs and the translocation of NF-κB from the cytosol to the nucleus, and then leads to the induction of various downstream target genes. Thus, the effect of GHRP-2 on MAPKs phosphorylation and NF-κB translocation was monitored in KGN cells. It was noted that PDD was able to induce p38 and JNK phosphorylation at 15 min (Figure 7A,B) and ERK phosphorylation at 15 and 30 min (Figure 7C); GHRP-2 appeared to reduce PDD-induced p38 and JNK phosphorylation at 15 min (Figure 7A,B), but it was not able to affect PDD-induced ERK phosphorylation (Figure 7C). The MAPKs p38 and JNK pathways have been shown to result in activator protein 1 (AP-1)-dependent gene expression [37]. Therefore we further investigated the regulation of AP-1 reporter activity by PDD and GHRP-2 in KGN cells. GHRP-2 alone did not affect AP-1 reporter activity, but PDD treatment did induce AP-1 reporter activity and such induction was abrogated by GHRP-2 (Figure 7D). In addition, we also determined the impact of GHRP-2 on PDD-mediated NF-κB activation in KGN cells. PDD treatment for 60 min significantly increased p65 translocation from the cytosol to the nuclear compartment (Figure 8A), while PDD in combination with GHRP-2 down-regulated p65 nuclear translocation (Figure 8A). We also further examined the regulation of NF-κB reporter activity in the presence/absence of PDD and GHRP-2. It was apparent that GHRP-2 alone did not affect NF-κB reporter activity, but PDD was able to induce NF-κB reporter activation and such induction was suppressed by GHRP-2 (Figure 8B).

### 2.5. Involvement of MKP-1 and PP2A in the Inhibitory Effect of GHRP-2 on PKC-Mediated COX-2 and IL-8 Expression

**Figure 9:**
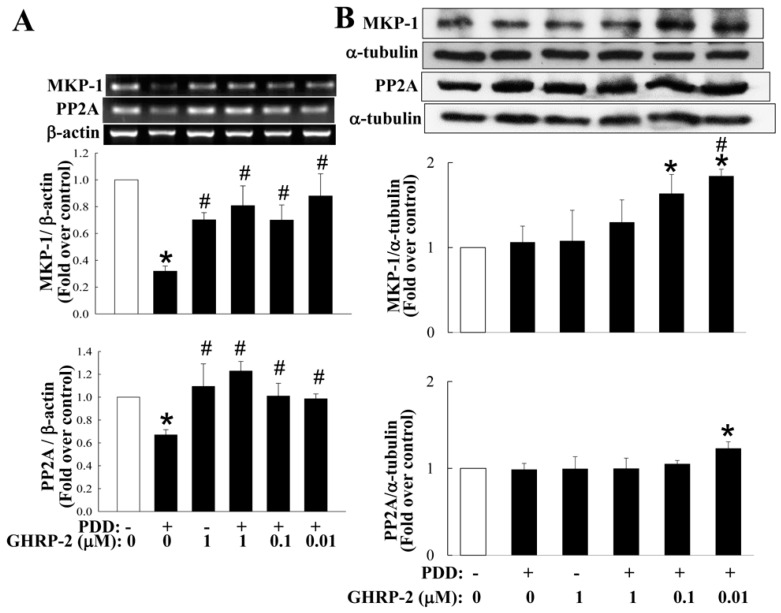
Regulation of mitogen-activated protein kinase phosphatase-1 (MKP-1) and protein phosphatase 2A (PP2A) expression by PDD and GHRP-2. Plated KGN cells were pretreated with GHRP-2 (0.01, 0.1, 1 µM) for 2 h, and PDD (100 nM) was included for an additional 12 h. The MKP-1 and PP2A mRNA or protein expression levels were determined by RT-PCR (**A**) and Western blotting assay (**B**), respectively. The results represent the means ± SEM ((**A**): *n* = 3; (**B**): *n* = 4) separate experiments. * *p* < 0.05 compared with the control; ^#^ *p* < 0.05 compared with the PDD treatment.

**Figure 10:**
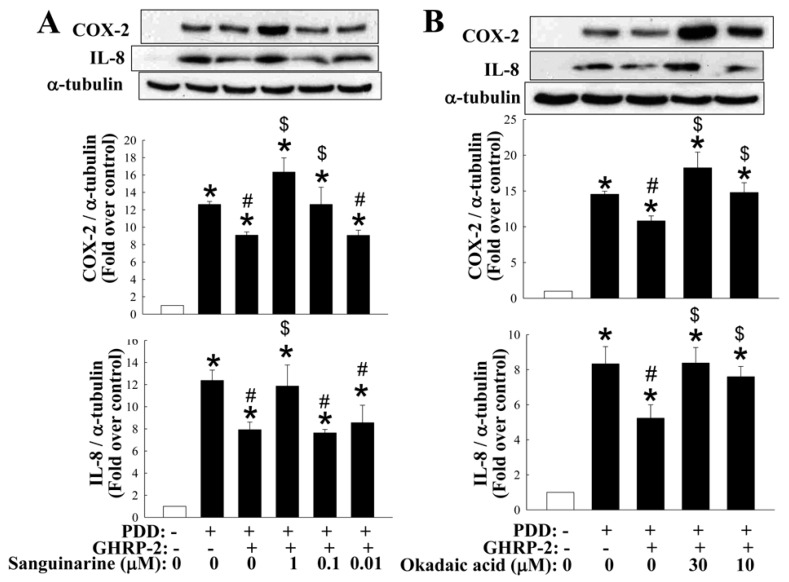
Involvement of MKP-1 and PP2A in the inhibitory effect of GHRP-2 on PKC-mediated COX-2 and IL-8 expression. To test the involvement of MKP-1 or PP2A in GHRP-2 inhibition of PKC-induced COX-2 and IL-8 expression, plated KGN cells were pretreated with GHRP-2 (1 µM) or a combination of GHRP-2 with either the MKP-1 inhibitor sanguinarine (0.01, 0.1, and 1 µM) (**A**) or the PP2A inhibitor okadaic acid (10, and 30 µM) (**B**) for 2 h, and then PDD (100 nM) was included for an additional 12 h. The intracellular COX-2 and IL-8 protein expression levels were monitored by Western blotting assay. The results represent the means ± SEM (*n* = 4) separate experiments. * *p* < 0.05 compared with the control; ^#^ *p* < 0.05 compared with the PDD treatment; ^$^ *p* < 0.05 compared with the combined GHRP-2 and PDD treatment.

The MAPKs pathways have been documented to be modulated by phosphorylation via the action of kinases or via dephosphorylation by phosphatases [38]. It has also been shown that the MAPK phosphatases (MKPs) are able to act as negative regulators of the MAPK pathways [39]. More than ten MKPs have been identified in mammalian cells and in particular, MKP-1 has been shown to dephosphorylate p38 and JNK [40], and thus may play a role in attenuating inflammatory response [41]. In addition, protein phosphatases have also been reported to be important during the regulation of NF-κB activation [42]. For example protein phosphatase 2A (PP2A) has been shown to directly dephosphorylate IκB and consequently attenuate the activation of NF-κB [43]. Thus, we next investigated whether the inhibitory effect of GHRP-2 on MAPK-mediated and NF-κB-mediated COX-2 and IL-8 expression also involves an interaction with either MKP-1 or PP2A. Firstly, we found that both MKP-1 and PP2A mRNA expression seemed to be inhibited by PDD as compared with the control treatment, while GHRP-2 was able to reverse the PDD suppression of MKP-1 and PP2A mRNA expression (Figure 9A). With the same treatments, we also monitored the protein expression of MKP-1 and PP2A and surprisingly it appeared that PDD did not affect the protein expression of either, but a lower dose of GHRP-2 was able to induce protein expression of both MKP-1 and PP2A (Figure 9B). To further examine whether MKP-1 and PP2A may act downstream of GHRP-2 during the anti-inflammatory effect, KGN cells were pretreated with GHRP-2 (1 µM) or with GHRP-2 in combination with an inhibitor, either a MKP-1 inhibitor (sanguinarine; 0.01, 0.1, and 1 µM) or a PP2A inhibitor (okadaic acid; 10, and 30 µM) for 2 h before the addition of PDD for an additional 12 h. Sanguinarine at 0.1 or 1 µM was able to attenuate the suppression effect of GHRP-2 on PDD-induced COX-2 expression (Figure 10A) and at 1 µM, it was also able to attenuate the inhibitory effect of GHRP-2 on PDD-induced IL-8 expression (Figure 10A). Similarly, okadaic acid at 10 or 30 µM significantly reversed the inhibitory effect of GHRP-2 on PDD-induced COX-2 and IL-8 expression (Figure 10B).

### 2.6. Involvement of the Akt Pathway in the Regulation by GHRP-2 of the PKC-Mediated Production of COX-2 and IL-8

**Figure 11:**
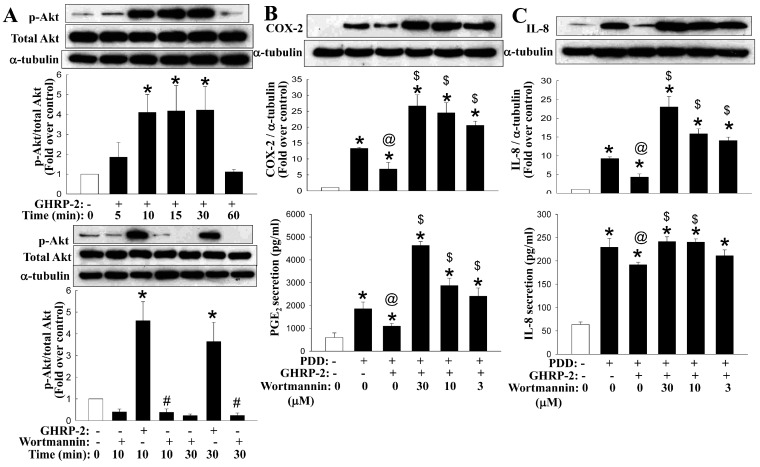
Involvement of the PI3K-Akt pathway in regulation by GHRP-2 of the PKC-mediated production of COX-2 and IL-8. (**A**) To examine whether Akt is under GHRP-2 regulation, plated KGN cells were treated with to GHRP-2 (1 µM) alone for 5, 10, 15, 30 or 60 min or with GHRP-2 in combination with wortmannin (10 µM) for 10 or 30 min. The Akt phosphorylation was monitored by Western blotting assay. (**B**,**C**) To evaluate the role of Akt in the GHRP-2 regulation of PKC-induced inflammation, plated KGN cells were pretreated with GHRP-2 (1 µM) alone or in combination with the PI3K-Akt inhibitor wortmannin (3, 10, and 30 µM) for 2 h, and then PDD (100 nM) was included for an additional 12 h. The intracellular COX-2 (**B**) and IL-8 (**C**) protein expression levels and the resulting PGE_2_ (**B**) and IL-8 (**C**) concentrations in the cultured media were determined by Western blotting assay and ELISA, respectively. The results are expressed as means ± SEM. ((**A**): *n* = 3; (**B**,**C**): *n* = 4). * *p* < 0.05 compared with the control treatment; ^#^ *p* < 0.05 compared with the GHRP-2 treatment group at the same time point; ^@^ *p* < 0.05 compared with the PDD treatment; ^$^ *p* < 0.05 compared with the combined GHRP-2 and PDD treatment.

Previously, GHRP-2 has been demonstrated to activate the PI3K-Akt signaling pathway, bringing about a cellular effect [44]. Thus, we next evaluated whether the inhibitory effect of GHRP-2 on PKC-mediated COX-2 and IL-8 expression might be mediated via the PI3K-Akt signaling pathway. Firstly, the effect of GHRP-2 on Akt activation (phosphorylation) was monitored at 5, 10, 15, 30, or 60 min. GHRP-2 appeared to induce Akt phosphorylation at 10, 15, and 30 min (Figure 11A), and this Akt phosphorylation was found to be inhibited by the PI3K inhibitor wortmannin (Figure 11A). In addition, treatment of KGN cells with wortmannin (3, 10, and 30 µM) apparently neutralized the suppression effect of GHRP-2 on PDD-induced COX-2 and IL-8 expression (Figure 11B,C). In addition, wortmannin at all doses was able to reverse the GHRP-2’s suppression effect on PDD-mediated PGE_2_ secretion (Figure 11B) and at 10 or 30 µM, it also reversed the GHRP-2’s suppression effect on PDD-induced IL-8 secretion (Figure 11C).

### 2.7. Inhibition of PKC-Induced COX-2 Expression by GHRP-2 in Primary Rat Ovarian Granulosa Cells

To confirm the role of ghrelin (GHRP-2) in ovarian inflammation using primary ovarian granulosa cells, we also evaluated the effect of GHRP-2 on rat primary ovarian granulosa cells. It was found that GHRP-2 at 0.1 or 1 µM appeared to attenuate the PDD-induced expression of COX-2. In this context GHRP-2 (1 µM) alone had no effect on COX-2 expression (Figure S5).

## 3. Discussion

This in vitro study mimics the inflammation microenvironment within the ovary and allows the investigation of the potential inhibitory impact of a ghrelin analog GHRP-2 on human granulosa cells using a PKC-induced local production of two inflammation mediators COX-2 and IL-8. Our results demonstrate that promoter activity, mRNA expression and protein expression of the COX-2 and IL-8 genes are all induced by the PKC activator PDD. GHRP-2 would seem to target the PKC-mediated activation of MAPKs p38, JNK, and NF-κB, as well as Akt, and this subsequently reduces the PKC-mediated induction of COX-2 and IL-8 production. MKP-1 and PP2A seemed to act downstream of GHRP-2 within an anti-inflammatory activity under conditions where PKC is activated.

Two GHSRs, namely GHSR-1a and GHSR-1b, have been identified [45] and we found that that only GHSR1a is detected in KGN human granulosa cells (Figure S2). Our findings are in accordance with previous studies that intense GHSR-1a immunoreactivity was noted in the granulosa cells of developing follicles [16,46], suggesting an important role for ghrelin and GHSR-1a in the ovary [47]. Some previous studies have indicated that ghrelin has an anti-inflammation effect. For examples, ghrelin has been shown to inhibit tumor necrosis factor (TNF-α)-induced IL-8 production in human endothelial cells [48] and the angiotensin II-induced expression of TNF-α, IL-8 and monocyte chemoattractant protein-1 (MCP-1) in human umbilical vein endothelial cells [49]. Furthermore, the ghrelin analog GHRP-2 has been shown to exert an antioxidant effect both in vivo and in vitro, but it does not seem to have any anti-atherogenic impact [50]. Similarly, GHRP-2 administration has been demonstrated to inhibit LPS-induced liver inflammation and endotoxemia in rats [29], as well as Freund's adjuvant-induced inflammation in arthritic rats [30]. In contrast to the above findings, it has also been reported that ghrelin is able to induce COX-2 expression and prostaglandin E_2_ production in human colonic epithelial cells [51]. Nevertheless, in the present study, we observed a clear anti-inflammatory effect of GHRP-2 on PKC-induced inflammation using KGN human ovarian granulosa cells (Figure 1). Involvement of the cognate receptor of ghrelin or GHRP-2, namely GHSR-1a was confirmed by treatment with a selective GHSR-1a antagonist JMV3002 (Figure 2). These findings provide good support for the hypothesis that ghrelin (GHRP-2) acts as an anti-inflammatory player via GHSR-1a in the ovarian system.

When considering the cellular target sites required for a molecule to modulate agonist-induced cytokine expression and subsequent secretion, there is a panel of potential sites. In this study, we have addressed the importance of ghrelin (GHRP-2) to PKC-mediated production of COX-2 and IL-8. In fact, several signaling pathways, including the various MAPKs and NF-κB, are known to be involved in regulating COX-2 [52,53] and IL-8 expression [5,54]. In this study we demonstrated that the PKC-mediated COX-2 expression involves, at least in part, all of the MAPKs (p38, ERK, JNK) as well as NF-κB, while IL-8 expression involves, at least in part, all of the MAPKs pathways but not the NF-κB (Figure 6). In addition, we have also shown that GHRP-2 may potentially target to p38, JNK, and NF-κB in order to down-regulate PDD-induced phosphorylation of p38 and JNK (Figure 7A,B), AP-1 reporter activation (Figure 7D) and PDD-activated NF-κB nuclear translocation and reporter activation (Figure 8). In fact, the involvement of the AP-1 and NF-κB responsive elements in the regulation of the COX-2 [53,55] and IL-8 [56,57] promoters has been reported previously, which supports our findings that AP-1 and NF-κB are two important signaling targets involved in the anti-inflammatory function of GHRP-2 during the PKC-induced transcription of COX-2 and IL-8. Interestingly, ERK phosphorylation was induced by GHRP-2 (Figure 7C). This is in accordance with a previous study showing that ghrelin is able to induce ERK phosphorylation during cell proliferation [58]. Furthermore, previous studies have demonstrated that MKP-1 is able to inactivate the MAPKs p38 and JNK pathways [59] and that PP2A is able to inactivate the NF-κB pathway [60]. Consistently with these two reports, we found that, although PDD did not affect the protein level of either MKP-1 or PP2A, GHRP-2 is able to in fact up-regulate both of them (Figure 9B). Why the impacts of PDD and GHRP-2 on mRNA and protein levels of MKP-1 and PP2A appear different and the lower dose, but not the higher dose of GHRP-2 is more effective are currently a mystery to us and will need further investigation. Previous studies have reported that ghrelin has an inhibitory effect on sepsis-induced inflammation via the up-regulation of MKP-1 expression [41], and the administration of a GHSR-1a agonist to aging mice has been shown to restore a young liver phenotype; the latter occurs via an increase in PP2A activity [61]. In the present study we have further noted that inhibitors of MKP-1 and PP2A are able to neutralize the suppression effect of GHRP-2 on PKC-induced COX-2 and IL-8 expression (Figure 10A,B). All of these findings, when taken together, strongly support the hypothesis that, in human granulosa cells, GHRP-2 (ghrelin) is able to inactivate the p38 and JNK pathways and NF-κB signaling by acting via the MKP-1 and PP2A, resulting in an attenuation of PKC-mediated COX-2 and IL-8 production.

It has been previously reported that ghrelin seems to activate the PI3K-Akt signaling pathway by interacting with its cognate receptor GHSR-1a [62]. Indeed in our study, GHSR-1a was detected in KGN human granulosa cells (Figure S2) and GHRP-2 was able to induce Akt phosphorylation (Figure 11A), and the GHRP-2’s inhibitory effect on PKC-induced inflammation in terms of COX-2 and IL-8 production was dramatically suppressed by the PI3K-Akt inhibitor wortmannin (Figure 11B,C). Both lines of evidence support the idea that Akt signaling may act downstream of GHRP-2 to perform the anti-inflammatory features. In fact, similar to our findings, it has been reported previously that, using the human embryonic kidney 293 cell line, ghrelin is able to induce Akt phosphorylation [44]. This also supports the hypothesis that the use of GHRP-2 in our study is an appropriate way of mimicking the action of ghrelin in the ovarian system.

Down-regulation of COX-2 and IL-8 proteins has also been reported to be regulated at the post-translational stage [8]. Intriguingly as shown in the current study, the degradation pattern of IL-8 appears to be different from that of COX-2, as the expression of COX-2 involved as lower down-regulation over time, but, nevertheless, GHRP-2 was able to significantly accelerate COX-2 degradation (Figure 3); although GHRP-2 did accelerate IL-8 protein degradation, IL-8 could be seen suddenly to disappear as early as 1 h and 3 h, and then bounced back with time (Figure 3). At this moment, we have no solid interpretation to explain this phenomenon. However, intriguingly, there are some previous articles reporting the novel effect of CHX on transcription and stability of IL-8 mRNA. In human neutrophils (PMNs), the degradation of IL-8 mRNA was shown to be finely regulated and CHX treatment was able to superinduce the IL-8 mRNA accumulation in a dose- and time-dependent manner [63]. In fact, in human hepatoma Huh7 cells, it was demonstrated that IL-8 and several other inflammatory genes all contain the AU rich elements (AREs) in the 3’-untranslated region (3’-UTR), which potentially plays an important role to regulate the mRNA stability; CHX together with TNF-a were noted to perform a superinduction effect of mRNAs of IL-8 and these inflammatory genes [64]. Besides the mRNA stability aspect, in lung epithelial H292 cells, CHX was also shown to enhance IL-8 mRNA transcription with the involvement of two important responsive elements AP-1 and NF-κB, resulting in IL-8 mRNA superinduction [65]. In HL-60 promyelocytic leukemia cell line, IL-8 mRNA was rapidly induced at high levels by PKC activator phorbol 12-myristate 13-acetate and unidentified negatively-acting transcriptional regulator(s) was suggested to involve in the modulatory effect of CHX on IL-8 mRNA induction [66], and consistently in human PMN, the IL-8 mRNA superinduction effect by CHX was also proposed to be due to its ability to prevent the de novo protein synthesis of NRF, a protein shown to repress IL-8 mRNA synthesis [67]. Interestingly in human keratinocytes, it was reported that CHX, but not another protein synthesis inhibitor puromycin, was able to induce IL-8 mRNA expression [68]. Therefore, in our study for monitoring the IL-8 protein stability (Figure 3), as PDD can rapidly induce IL-8 transcription, and although the PDD was removed before the inclusion of CHX in our study, there might be still some minor amount of PDD remaining inside the cells and by acting together with CHX, IL-8 mRNA was then superinduced by promoting either transcription or stabilization or both. The CHX treatment may not completely block the translation machinery 3 h later, and thus the accumulated superinduced IL-8 mRNA can then be translated into IL-8 protein. According to our findings, PDD in combination with the proteasome inhibitor MG132 is able to enhance IL-8 expression compared to PDD alone treatment (Figure 4B), which suggests a possibility that CHX treatment is able to target the proteasome pathway leading to degradation of IL-8 protein. However, this speculation needs more in-depth investigation to solve this puzzle. Regardless of the above anomalies, our findings clearly demonstrated that GHRP-2 promoted degradation of the PKC-induced COX-2 and IL-8 proteins; these phenomena can be reversed by the proteasome inhibitor MG132 or the lysosome inhibitor chloroquine (Figure 4). These findings suggest that GHRP-2 may interact with both the proteasome and lysosome pathways to mediate the degradation of the COX-2 and IL-8 proteins in human granulosa cells.

In addition to our studies using human granulosa cells, we also evaluated the anti-inflammatory effect of GHRP-2 on PDD-mediated COX-2 expression in rat ovarian granulosa cells. Consistent with our findings in human granulosa cells, GHRP-2 appeared to significantly attenuate PDD-induced COX-2 expression (Figure S5), which supports the anti-inflammatory role of ghrelin (GHRP-2) in the granulosa cells of both humans and rats. The use of cell lines to mimic the in vivo physiology is a good tool; however, the cell lines may not act identically as the primary cells within the body. For example, the KGN cell line used in this study was originally from a 63-years old woman [69] and thus it may not represent the same granulosa cells from the other aged ovaries. Regardless, in addition to our current findings of ghrelin in ovarian inflammation, previous studies have already revealed various impacts of ghrelin in the reproductive system [19,20,21,22,23,24,25,26,27]. Thus, there is a possibility that ghrelin administration might be a potential strategy to treat inflammation-related diseases in reproductive tissues.

**Figure 12:**
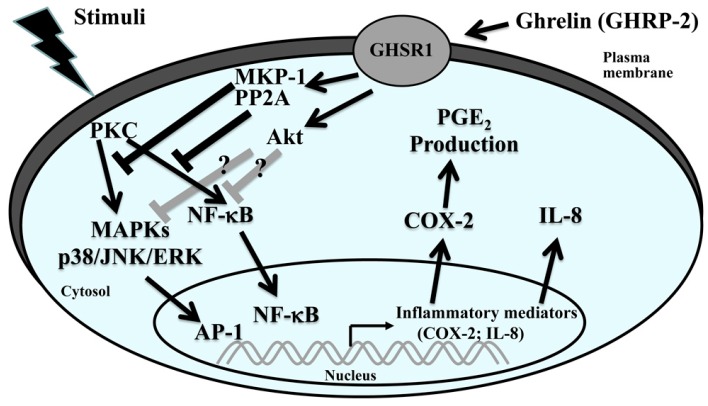
Outline of the PKC regulation of COX-2 and IL-8 production and the potential acting points of ghrelin (GHRP-2) in ovarian granulosa cells. PKC activates the intracellular signaling pathways, including the MAPKs (p38, JNK, ERK) as well as the NF-κB pathways, both of which would lead to transcription of COX-2 and IL-8, and consequently mediate the output of PGE_2_ and IL-8. Ghrelin (GHRP-2) may regulate MKP-1 and PPA2 to affect the PKC-mediated activation of MAPKs and NF-κB, as well as activate the Akt pathway, and subsequently to attenuate the PKC-induced COX-2 and IL-8 transcription, resulting in reduction of COX-2 and IL-8 protein levels as well as PGE_2_ and IL-8 secretion.

In conclusion, our study reveals a novel role for GHRP-2 as a potent anti-inflammation molecule that is able to impact ovary inflammation in vitro. Our findings highlight a new role for the anti-inflammatory molecule ghrelin in the ovarian granulosa cells that involves control of PKC-mediated COX-2 and IL-8 expression and the secretion of PGE_2_ and IL-8. These effects of GHRP-2 occur, at least in part, by targeting to a number of signaling cascades involving various MAPKs, NF-κB, and Akt (Figure 12).

## 4. Experimental Section

### 4.1. Chemicals and Reagents

Phorbol 12, 13-didecanoate (PDD) was purchased from Enzo Life Sciences (Farmingdale, NY, USA). Bisindolylmaleimide I (BIM I, a PKC inhibitor) was purchased from Cayman Chemical (Ann Arbor, MI, USA). Fetal bovine serum was obtained from HyClone (Logan, UT, USA). Reverse transcriptase and Taq polymerase were purchased from Promega (Madison, WI, USA). The mouse monoclonal antibody against human IL-8 was purchased from R&D systems. The mouse monoclonal antibody against COX-2 was purchased from Cayman Chemicals. The rabbit polyclonal antibodies against p65 were purchased from Neomarkers (Fremont, CA, USA). The mouse monoclonal antibody against MKP-1 was from Abnova (Taipei, Taiwan). The goat polyclonal antibody against PP2A and the mouse monoclonal antibody against histone H1 were purchased from Santa Cruz Biotechnology (Santa Cruz, CA, USA). The mouse monoclonal antibody against α-tubulin and the horseradish peroxidase-conjugated donkey anti-rabbit IgG secondary antibodies were purchased from Amersham Life Science Inc. (Arlington Heights, IL, USA). Unless otherwise specified, all other chemicals and reagents used in this project were from Sigma.

### 4.2. Cell Culture

The immortalized human granulosa cell line KGN [69] was purchased from the RIKEN BioResource Center (Iberaki, Japan) and was maintained using Dulbecco’s modified Eagle medium: nutrient mixture F-12 (Ham) (1:1) (DMEM/F-12) with 10% fetal bovine serum (FBS), 2 g/L sodium bicarbonate, 100 U/mL penicillin and 100 µg/mL streptomycinin an atmosphere of 5% CO_2_ at 37 °C.

### 4.3. Western Blotting Analysis

Protein extracts of total cellular proteins and of nuclear proteins were harvested and their protein concentrations were determined using the Bio-Rad protein assay reagent (Bio-Rad, Hercules, CA, USA), and the total protein concentration was adjusted with SDS-PAGE loading buffer and heated to 100 °C for 10 min and then subject to regular Western blotting assay to determine the expression profile of various proteins. Samples of equal amounts of proteins (50 µg) were separated on 10% SDS-PAGE, transferred onto a nitrocellulose membrane, blocked with 5% milk for 1 h, and incubated overnight with various specific antibodies, followed by incubation for 2 h with the corresponding horseradish peroxidase-coupled secondary antibodies. The membrane was exposed to film and the bands of interest on the film were quantified with ImageQuant 5.2 software (Molecular Dynamics, Sunnyvale, CA, USA).

### 4.4. Enzyme-Linked Immunosorbent Assay (ELISA)

The concentrations of IL-8 and PGE_2_ in the culture medium were determined using enzyme-linked immunosorbent assay kits for IL-8 (R&D systems, Minneapolis, MN, USA) and PGE_2_ (Assay Designs, Ann Arbor, MI, USA) according to the manufacturers’ instructions.

### 4.5. Semi-Quantitative Reverse Transcription Polymerase Chain Reaction (RT-PCR)

The total cellular RNAs were extracted using Tri-Reagent (Sigma) according to the manufacturer's instructions. The isolated RNA samples were resuspended in RNase-free diethylpyrocarbonate (DEPC)-treated water and followed by a regular two-step semi-quantitative RT-PCR method to examine the levels of various mRNAs, namely those encoding COX-2, IL-8, GHSR-1a, BRCA1, Cathepsin D, MKP-1, PP2A, GAPDH, and β-actin. In brief, 1 µg of total RNAs from each sample was used to perform the reverse transcription, and to detect the cDNA contents, 2 µL cDNA from RT reaction was added into the PCR reaction tube and mixed with 10× PCR buffer, 0.5 mM dNTP, 0.5 µM sense and antisense primers (MDBio Inc., Taipei, Taiwan), and 0.2 U TaqDNA polymerase, using a Program Temp Control System PC 818 (Astec Technology, Fukuoka, Japan). The primer sequences used are listed in Table 1. The PCR products were analyzed by electrophoresis in 2% agarose gel with 1 µg/mL ethidium bromide. The final cDNA yields were then determined from the amplified DNA signals by comparing them against the internal control GAPDH or β-actin. The DNA signals were captured and analyzed by ImageQuant 5.2 software.

### 4.6. Transfection and Analysis of COX-2 and IL-8 Promoter Activity Levels as well as NF-κB and Activator Protein-1 (AP-1) Reporter Activity Levels

Transfection was performed using Lipofectin reagent (Invitrogen, Paisley, UK) according to the manufacturer’s instructions. To examine the levels of COX-2 and IL-8 promoter activity, plated KGN cells were transfected with either a COX-2 [70] or an IL-8 promoter construct (a gift from Dr. N Mukaida, Kanazawa University, Kanazawa, Japan). To analyze activation of the NF-κB and AP-1 promoters, either a minimal promoter sequence bearing multiple NF-κB binding sites and driving a luciferase reporter gene (a gift from Dr. Bing-Chang Chen, Taipei Medical University, Taipei, Taiwan) or a reporter plasmid with AP-1 responsive elements fused to a luciferase reporter gene was separately transfected into KGN cells. During all of these experiments, a pCMV-β-Gal plasmid was co-transfected as a control.

### 4.7. Statistical Analysis

Experimental data are expressed as means plus/minus the standard errors of the means (mean ± SEM). The results were analyzed by one-way analysis of variance (ANOVA), which was followed by the least-significant difference (LSD) test; this approach was used to compare the differences between the various treatment groups and the control groups. Differences with a *p* value of less than 0.05 were considered to be statistically significant.

## Figures and Tables

**Table 1:** Oligonucleotide primers for RT-PCR.

Gene	Sequences	Direction	Size (bp)
*COX-2*	5′-GCATCAGTTTTTCAAGACAG-3′ 5′-TCGCATACTCTGTTGTGTTC-3′	Sense Antisense	324
*IL-8*	5′-ACTTCCAAGCTGGCCGTGGCT-3′ 5′-TCACTGGCATCTTCACTGATT-3′	Sense Antisense	318
*PP2A*	5′-AAGGTTCGTTACCGTGAACG-3′ 5′-ACCTCTTGCACGTTGGATTC-3′	Sense Antisense	641
*MKP-1*	5′-CCGGAGCTGTGCAGCAAA-3′ 5′-CTCCACAGGGATGCTCTT-3′	Sense Antisense	282
*GHSR-1a*	5′-AGCGCTACTTCGCCATC-3′ 5′-CCGATGAGACTGTAGAG-3′	Sense Antisense	289
*GHSR-1b*	5′-TCTTCCTTCCTGTCTTCTGT-3′ 5′-GATAGGACCCGCGAGAGAAA-3′	Sense Antisense	179
*β-actin*	5′-GGCACCACACCTTCTACAAT-3′ 5′-CGTCATACTCCTGCTTGCTG-3′	Sense Antisense	834
*GAPDH*	5′-ATCACCATCTTCCAGGAGCG-3′ 5′-CCTGCTTCACCACCTTCTTG-3′	Sense Antisense	574
*BRCA1*	5′-ACAGCTGTGTGGTGCTTCTGTG-3′ 5′-CATTGTCCTCTGTCCAGGCATC-3′	Sense Antisense	107
*Cathepsin D*	5′-CATTGTGGACACAGGCACTTC-3′ 5′-GACACCTTGAGCGTGTAGTCC-3′	Sense Antisense	201

## Supplementary Materials

Supplementary materials can be found at www.mdpi.com/1422-0067/17/8/1359/s1.
