# Supplementary material for: Growth Hormone Releasing Peptide-2 Attenuation of Protein Kinase C-Induced Inflammation in Human Ovarian Granulosa Cells
Source: Int J Mol Sci. 2016 Aug 19;17(8):1359. doi: 10.3390/ijms17081359 (PMC5000754; doi:10.3390/ijms17081359)
Supplement: Supplementary file 1 [file ijms-17-01359-s001.pdf]

Supplementary Materials: Growth Hormone Releasing Peptide-2 Attenuation of Protein Kinase C-induced Inflammation in Human Ovarian Granulosa Cells

Yi-Ning Chao, David Sun, Yen-Chun Peng and Yuh-Lin Wu

Isolation of Rat Ovarian Granulosa Cells

Female Sprague-Dawley rats weighing between 200 and 300 g were allowed to acclimatize for one week to being housed at 25 °C on a 12-h light/12-h dark cycle; all animals were allowed free access to water and standard laboratory chow. All animal care and experiments were approved by the Institutional Animal Care and Use Committee (IACUC) of National Yang-Ming University (IACUC Approval Number 1011217). Rat ovarian granulosa cells were isolated from immature female rats (26 days) after administration of pregnant mare's serum gonadotropin (PMSG). Fifteen IUs of PMSG were injected into each rat and, two days after injection, their ovaries were dissected and placed in L-15 medium with PSG (Penicillin, streptomycin, and gentamicin). The ovaries were punctured with a 25-gauge needle to release granulosa cells into the medium. Harvested granulosa cells were washed twice with PBS and centrifuged at 2500 rpm for 5 min. Approximately 4×10^6 cells were then seeded into a 3.5-cm dish in McCoy's 5A medium for overnight plating.

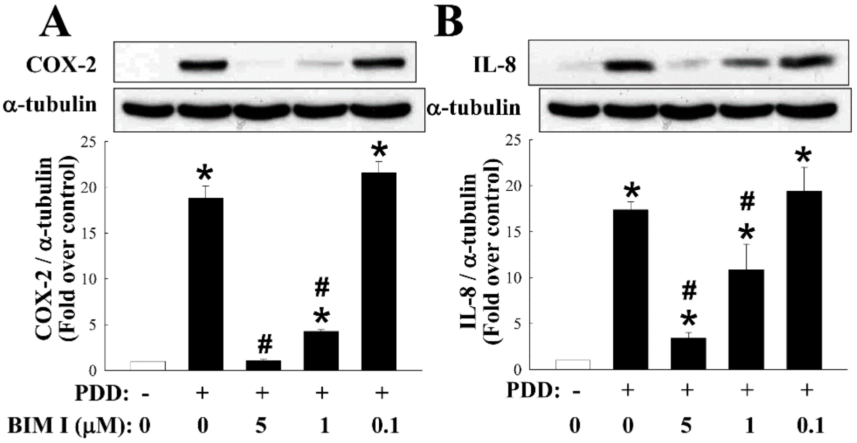

Figure S1. PKC-induced COX-2 and IL-8 expression in KGN cells. Overnight plated KGN cells were treated with PDD (100 nM) alone or in the presence of a PKC inhibitor bisindolylmalemide I (BIM I; 0.1, 1, 5 μM) for 12 h. The intracellular proteins levels of COX-2 (**A**) and IL-8 (**B**) were determined by Western blotting assay. The results represented the means ± SEM (n = 3) * $p < 0.05$ compared with the control; # $p < 0.05$ compared with the PDD treatment.

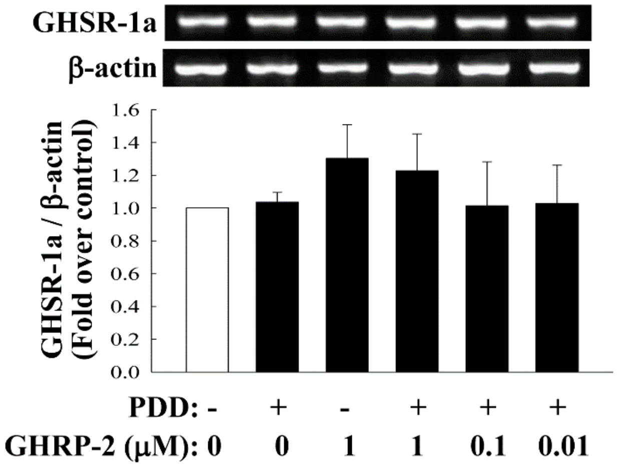

Figure S2. Expression of the ghrelin receptor GHSR type 1a in KGN cells. Overnight plated KGN cells were pretreated with GHRP-2 (0.01, 0.1, 1 μM) for 2 h, and then PDD (100 nM) was added for an additional 12 h. The GHSR-1a and GHSR-1b mRNA levels were determined by RT-PCR. The results represent the means ± SEM. (n = 3)

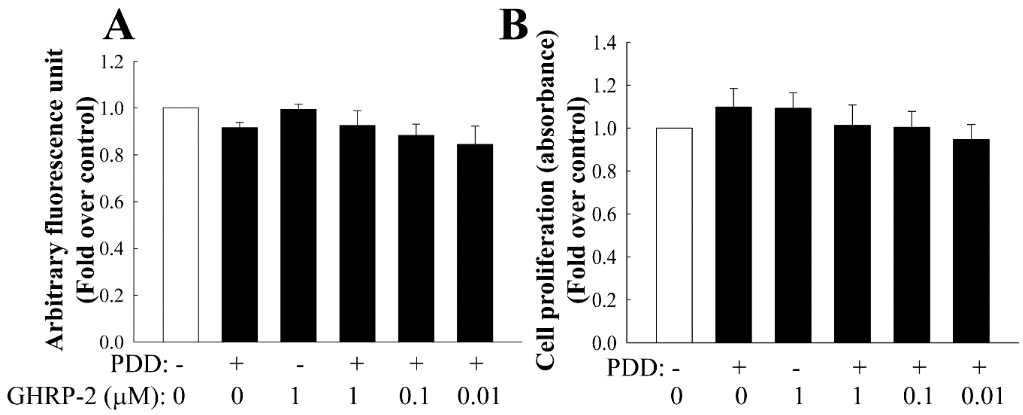

Figure S3. Non-cytotoxic effect of GHRP-2 on KGN cells. Overnight plated KGN cells were pretreated with GHRP-2 (0.01, 0.1, 1 μM) for 2 h, and then PDD (100 nM) was included for an additional 12 h. (**A**) For the alamarBlue assay, the conditioned medium was monitored at an excitation wavelength of 550 nm and an emission of 590 nm by spectrofluorometry; (**B**) For the MTT assay, cells were collected by treating with isopropanol then the color was quantified by reading the optical density at a test wavelength of 570 nm and a reference wavelength of 630 nm using an ELISA reader. The results represent the means ± SEM (n = 4)

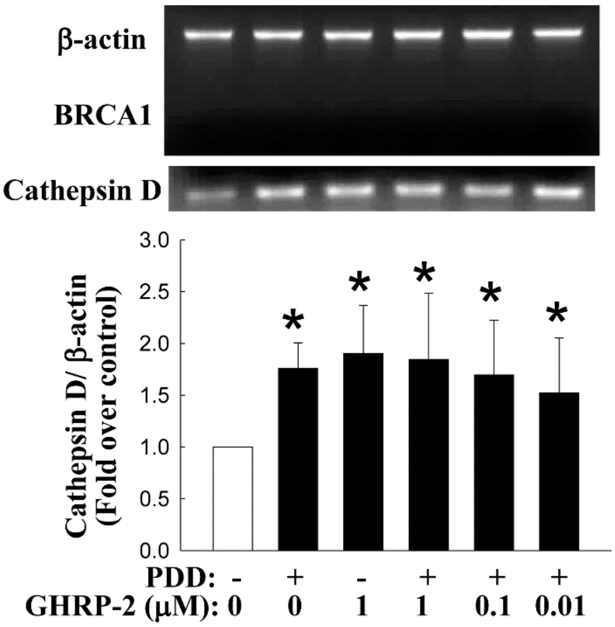

Figure S4. Expression of BRCA1 and Cathepsin D in KGN cells. Overnight plated KGN cells were pretreated with GHRP-2 (0.01, 0.1, 1 μM) for 2 h, and then PDD (100 nM) was included for an additional 12 h. The BRCA1 and cathepsin mRNA expression levels were determined by reverse transcriptase-PCR (RT-PCR). The results represent the means ± SEM (n = 3) * $p < 0.05$ compared with the control.

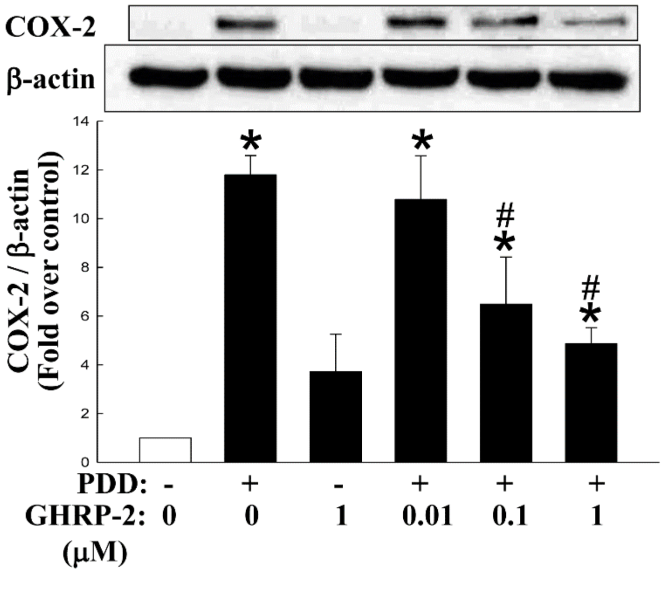

Figure S5. Inhibition of PKC-induced COX-2 expression by GHRP-2 in rat ovarian granulosa cells. Rat ovarian granulosa cells from pooled ovarian antral follicles were pretreated with GHRP-2 (0.01, 0.1, 1 μM) for 2 h, and then PDD (100 nM) was included for an additional 12 h. The intracellular COX-2 protein expression was assessed by Western blotting assay. The results represent the means ± SEM (n = 5) * $p < 0.05$ compared with the control; $^{\#}$ $p < 0.05$ compared with the PDD treatment.
